# How to fix the leaky pipeline in Academic Psychiatry

**DOI:** 10.1192/j.eurpsy.2023.60

**Published:** 2023-07-19

**Authors:** S. Frangou

**Affiliations:** ^1^University of British Columbia, Vancouver, Canada; ^2^Icahn School of Medicine at Mount Sinai, New York, United States

## Abstract

**Abstract:**

The leaky pipeline is a visual metaphor for the under-representation of women in leadership positions in Academic Psychiatry despite their over-representation in medical schools, residency programs, and junior academic positions. The presentation focuses on the key obstacles that pertain to personal and societal attitudes and institutional barriers and proposes empirically tested and pragmatic solutions.

**Disclosure of Interest:**

None Declared

